# Expression of Lipid-Metabolism Genes Is Correlated With Immune Microenvironment and Predicts Prognosis in Osteosarcoma

**DOI:** 10.3389/fcell.2021.673827

**Published:** 2021-04-16

**Authors:** Hu Qian, Ting Lei, Yihe Hu, Pengfei Lei

**Affiliations:** ^1^Department of Orthopeadic Surgery, Xiangya Hospital Central South University, Changsha, China; ^2^Hunan Engineering Research Center of Biomedical Metal and Ceramic Implants, Changsha, China; ^3^Department of Sports Medicine, Xiangya Hospital Central South University, Changsha, China

**Keywords:** osteosarcoma, lipid metabolism, immune microenvironment, prognosis, individualized therapy

## Abstract

**Objectives:**

Osteosarcoma was the most popular primary malignant tumor in children and adolescent, and the 5-year survival of osteosarcoma patients gained no substantial improvement over the past 35 years. This study aims to explore the role of lipid metabolism in the development and diagnosis of osteosarcoma.

**Methods:**

Clinical information and corresponding RNA data of osteosarcoma patients were downloaded from TRGET and GEO databases. Consensus clustering was performed to identify new molecular subgroups. ESTIMATE, TIMER and ssGSEA analyses were applied to determinate the tumor immune microenvironment (TIME) and immune status of the identified subgroups. Functional analyses including GO, KEGG, GSVA and GSEA analyses were conducted to elucidate the underlying mechanisms. Prognostic risk model was constructed using LASSO algorithm and multivariate Cox regression analysis.

**Results:**

Two molecular subgroups with significantly different survival were identified. Better prognosis was associated with high immune score, low tumor purity, high abundance of immune infiltrating cells and relatively high immune status. GO and KEGG analyses revealed that the DEGs between the two subgroups were mainly enriched in immune- and bone remodeling-associated pathways. GSVA and GSEA analyses indicated that, lipid catabolism downregulation and lipid hydroxylation upregulation may impede the bone remodeling and development of immune system. Risk model based on lipid metabolism related genes (LMRGs) showed potent potential for survival prediction in osteosarcoma. Nomogram integrating risk model and clinical characteristics could predict the prognosis of osteosarcoma patients accurately.

**Conclusion:**

Expression of lipid-metabolism genes is correlated with immune microenvironment of osteosarcoma patients and could be applied to predict the prognosis of in osteosarcoma accurately.

## Background

Osteosarcoma is the most common primary malignant bone tumor in children and adolescent, which is characterized by poor prognosis and high metastasis rate (Niu et al., 2020; Song et al., 2020). The incidence of osteosarcoma is related with the age of patients, which is about 0.0004% in individuals younger than 25 years old or older than 59 years old, while 0.0001% in individuals aged 25–59 years old (Whelan and Davis, 2018). It is worth noting that 15–20% patients had lung metastasis when they were first diagnosed with osteosarcoma (Miller et al., 2013). The 5-year survival rate of osteosarcoma patients with or without lung metastasis is 60–70%, and 20%, respectively, which has remained stagnant over the past 35 years and is far from satisfaction (Kansara et al., 2014; Negri et al., 2019).

Tracing back to the source, the main reason for the poor prognosis in osteosarcoma is the high extent of tumor heterogeneity caused by significant genomic instability (Whelan and Davis, 2018; Wang et al., 2019). Thus, it is necessary to develop a risk stratification method and identify prognostic genes for personalized targeted therapy of osteosarcoma patients.

Recently, lipid metabolism reprogramming has been regarded as a novel hallmark of tumor malignancy (Cheng et al., 2018), and increasing evidences from clinical and laboratory studies have revealed that lipid metabolism disorder plays a pivotal role in tumorigenesis, tumor progression and treatment (Corbet and Feron, 2017; Luo et al., 2017; Cao, 2019). Niemi et al. (2018) demonstrated that aberrant lipid metabolism in ovarian cancer intensified with increasing stage. Su et al. (2020) reported that enhanced lipid metabolism was necessary for the initiation and differentiation of tumor-associated macrophages. Hilvo et al. (2011) demonstrated that the lipids in the breast cancer tissue were correlated with tumor progression and patient survival. Moreover, previous studies have demonstrated that lipid metabolism related genes (LMRGs) had potent prognostic potential in multiple types of tumors, including ovarian carcinomas (Zheng et al., 2020), lung adenocarcinoma (LAUD) (Li et al., 2020), pancreatic cancer (Ye et al., 2021), hepatocellular carcinoma (HCC) (Hu et al., 2020), renal cell carcinoma (RCC) (Bao et al., 2019) and diffuse gliomas (Wu et al., 2019). As such, targeting lipid metabolism has been regarded as a novel therapeutic strategy for tumor treatment (Visweswaran et al., 2020). Construction of prognostic risk model was an applicable strategy to evaluate the prognostic performance. Up to now, several risk models have been constructed to explore the prognostic value of genes associated with tumor microenvironment, immune cell infiltrating and energy metabolism (Chen Y. et al., 2020; Wen et al., 2020; Zhang et al., 2020; Zhu et al., 2020) in osteosarcoma, whereas the role of LMRGs in osteosarcoma has remained poorly understood.

Tumor immune microenvironment (TIME), which reflexed the immune landscape in the tumor microenvironment, was essential for the initiation and development of tumors (Binnewies et al., 2018). Immune cells take part in the cell reprogramming critically, during which the microenvironment of the tumor cells was modified meticulously by themselves through secreting various kinds of biological factors, thereby endowing surrounding cells with powers to determine the survival and progression of tumors (Hinshaw and Shevde, 2019). In tumor microenvironment, tumor infiltrating immune cells account for the primary non-tumor constituents, which have been demonstrated to play an important role in prognostic prediction of OS patients (Zhang et al., 2020). Thus, TIME takes crucial significance in the development and progression of tumor, and accumulated evidence revealed that TIME was closely associated with pathogenesis of osteosarcoma (Heymann et al., 2019; Luo et al., 2020). Assessing the TIME of osteosarcoma helps to understand the immune status of tumor cells, is conducive to promote the development of immunotherapy and improve the prognosis of osteosarcoma patients.

In the present study, we comprehensively analyzed LMRGs to explore the effect of lipid metabolism on the TIME and survival of osteosarcoma patients. Moreover, we constructed a LMRGs-based risk score model to evaluate the prognostic value of LMRGs in osteosarcoma. Our work may provide a new clue for exploring the underlying molecular mechanisms of osteosarcoma, shed a novel light on the targeting therapy strategy of osteosarcoma and promote the individual-based treatment of osteosarcoma patients.

## Materials and Methods

### Data Collection

Clinical information and sequencing RNA data were downloaded from the Therapeutically Applicable Research To Generate Effective Treatments (TARGET^1^) and Gene Expression Omnibus (GEO^2^) databases. The inclusion criteria were as follows: (a) samples diagnosed as osteosarcoma; (b) samples with mapped clinical information and gene expression matrix; (c) samples with complete clinical information including survival time, survival status, age and sex at least; (d) only one was included if there were paired samples. The exclusion criteria were as follows: (a) normal tissue samples; (b) samples without complete clinical information; (c) samples with no expression value in over half of the genes; (d) samples with bias in expressional value. Ninety three samples acquired from the TARGET database were defined as the training cohort. Seventy samples acquired from GEO databases (GSE21257 and GSE39058) were included and defined as verification cohort after integrating. The demographic data and clinical features of the training cohort and validation cohort were listed in Table 1. Datasets of 776 LMRGs were obtained from the Reactome and KEGG databases.

**TABLE 1:** Characteristics of patients in the training and validation cohort.

	Training cohort (*n* = 93)	Validation cohort (*n* = 70)	*P*-value
		
	n/%	n/%	
Age			0.6115
<18 years	71/76.3	51/72.9	
>=18 years	22/30.1	19//27.1	
Sex			0.6649
Female	39/41.9	27/38.6	
Male	54/58.1	43/61.4	
Survival status			0.4311
Alive	55	47	
Dead	35	23	
Histologic response			0.0850
Stage 1/2	16	23	
Stage 3/4	27	16	
NA	47	31	
Race			
White	55	NA	
Asian	6	NA	
Black or African American	9	NA	
NA	20	NA	

*NA, not available.*

### Identification of Molecular Subgroups and TIME Evaluation

Firstly, 74 genes were found to be associated with the prognosis of osteosarcoma through the univariate Cox regression analysis. Consensus clustering was performed according to the expression matric of the 74 genes using the R package “ConsensusClusterPlus.” Stromal score, immune score, and tumor purity were calculated using the Estimation of Stromal and Immune cells in Malignant Tumor tissues using Expression data (ESTIMATE) algorithm (Yoshihara et al., 2013).

### Immune Analyses

TIMER immune infiltrating analysis^3^ was performed to calculate the abundance of six immune infiltrating cells (B cell, Macrophage cell, Dendritic cell, Neutrophil cell, CD4 T cell and CD8 T cell). Datasets including 28 types of immune infiltrating cells and related 782 genes were obtained from molecular signature database^4^, and the enrichment of the 28 immune infiltrating cells in the tumor samples were assessed using single sample gene set enrichment analysis (ssGSEA).

### Functional Analyses

Differentially expressed genes (DEGs) between the two clusters were identified using R package ‘‘Limma.’’ Gene Ontology (GO) analysis and Kyoto Encyclopedia of Genes and Genomes (KEGG) analysis were performed using ‘‘clusterProfiler’’ R package to enrich associated pathways, which was visualized in Metascape^5^. Based on “GO biological process” gene set downloaded from molecular signature database (see text footnote 5), Gene set variation analysis (GSVA) was performed to demonstrate the signaling pathways alteration between the two clusters using the “GSVA” R package. Meanwhile, according to the same dataset, Gene Set Enrichment Analysis (GSEA) was conducted to analyze the difference between clusters.

### Establishment and Validation of Risk Model

**FIGURE 1:**
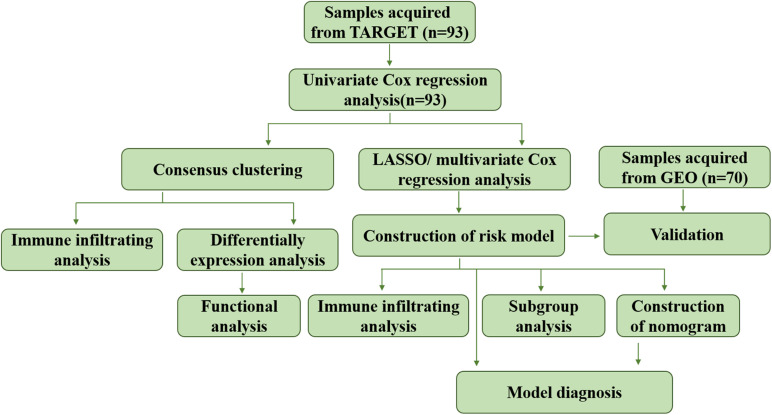
Flow chart of the data analyzing process.

Least absolute shrinkage and selection operator (LASSO) analysis was conducted to downsize the prognostic genes previously filtrated using “glmnet” R package. The minimum lambda was defined as the optimal value. The genes used for establishment of risk model was determined by multivariate Cox regression analysis. Risk score of each patient in the training and verification cohorts was calculated as: risk score = 1.7063 × expression value of *ALOX15B* + 0.7626 × expression value of *ME1* + 0.6200 × expression value of *GPD1*, and patients were divided into high risk and low risk groups according to the medium value. ROC and Martingale residuals method were performed to assess the predictive efficiency of the model. The whole process of data analysis was depicted in Figure 1.

### Statistical Analyses

Statistical analyses were performed via R (version 3.6.1) and GraphPad Prism (version 8.0.1), and visualization was conducted via TBtools (Chen C. et al., 2020). Survival analysis was completed using Kaplan–Meier method, and the prediction performance of the risk model was evaluated using time-dependent receiver operating characteristic (ROC) via “survivalROC” R package. Subgroup analysis was carried out when the patients were regrouped according to age, sex, lesion site and metastasis. Discontinuous data was presented as number/percentages while continuous data was shown in the form of mean ± standard deviation (SD). Student’s *t*-test was used for statistical analysis between two groups, and one-way ANOVA analysis was selected flexibly when there were three or more groups. A *P* < 0.05 was defined as statistically significant difference.

## Results

### Identification of Two Molecular Subtypes Based on LMRGs

**FIGURE 2:**
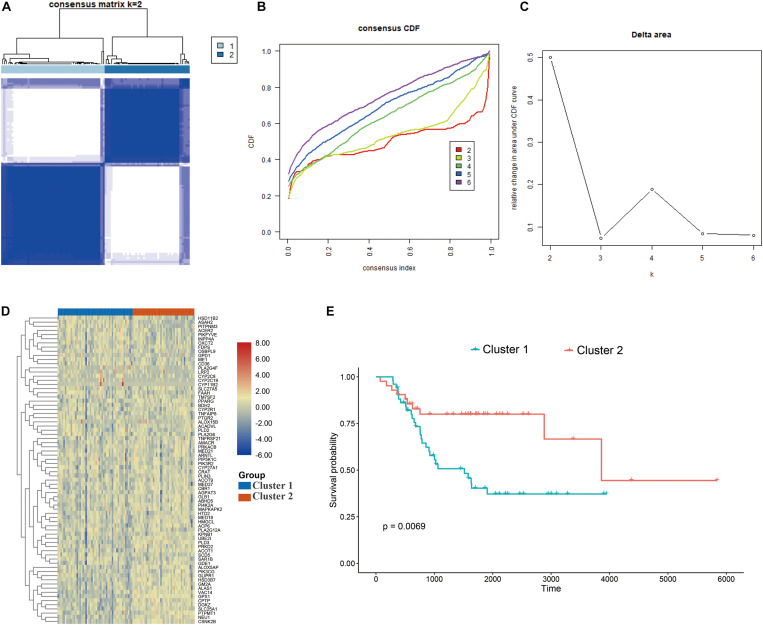
Consensus cluster. **(A–C)** *K* = 2 was identified the optimal value for consensus clustering, **(D)** heatmap visualizing the expression of lipid metabolism gens in the two subgroups, **(E)** survival curve of the patients in the two subgroups.

The consensus clustering approach was conducted to divide the osteosarcoma patients in the training cohort into subgroups based on 74 prognostic genes generated from univariable Cox analysis (Supplementary Table 1). The optimal clustering stability was identified when K = 2 (Figures 2A–C and Supplementary Figure 1). 51 patients were clustered into cluster 1 and 42 patients were clustered into cluster 2. The expression level of the LMRGs in the two subtypes was visualized through the heatmap (Figure 2D), and obvious expression difference was found between cluster 1 and cluster 2. Moreover, patients in the cluster 2 enjoyed better overall survival than patients in the cluster 1 (*P* = 0.0069; Figure 2E). These results demonstrated that the LMRGs classify the osteosarcoma patients into two molecular subtypes with different overall survival.

### Patients in the Two Molecular Subtypes Exhibited Different TIME and Immune Status

**FIGURE 3:**
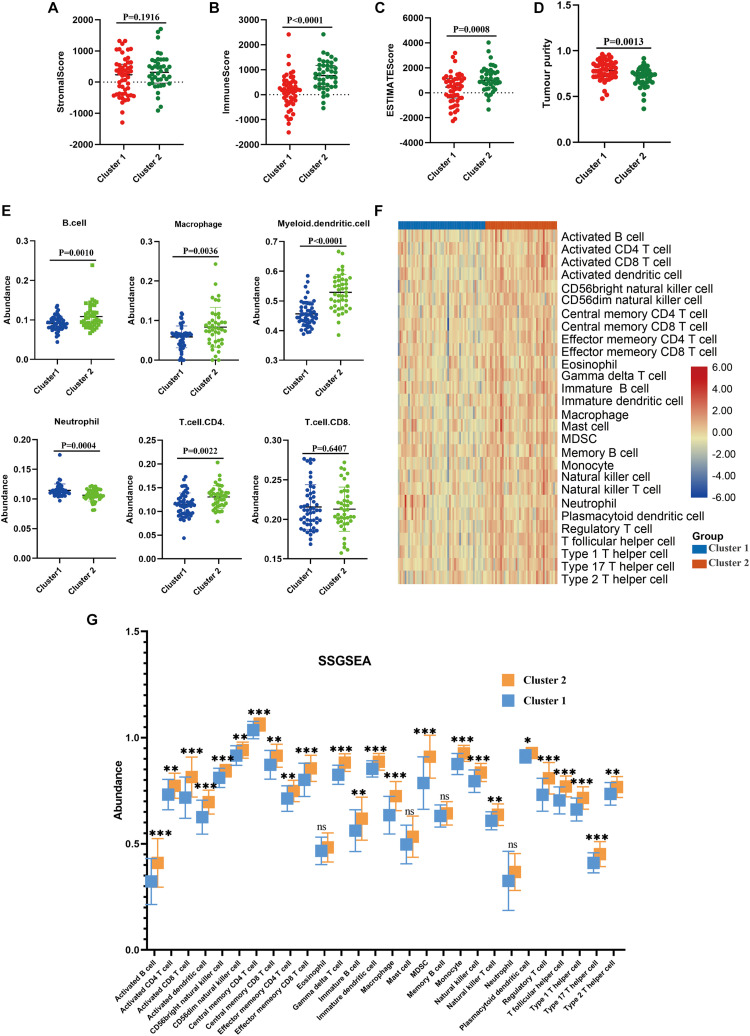
Immune analyses in the two clustered subgroups. **(A)** stromal score, **(B)** immune score, **(C)** ESTIMATE score and **(D)** tumor purity calculated by ESTIMATE algorithm, **(E)** abundance of six immune filtrating cells evaluated by TIMER, **(F)** heatmap depicting the enriching level of 29 immune related cells evaluated by ssGSEA algorithm, **(G)** statistical analysis of ssGSEA. *p < 0.05; **p < 0.01; ***p < 0.001.

Next, we performed immune analyses to explore the immune difference between the two molecular subtypes. ESTIMATE algorithm revealed that osteosarcoma patients in the cluster 2 had significantly higher immune score (*P* < 0.0001), ESTIMATE score (*P* = 0.0008) and lower tumor purity (*P* = 0.0013) compared with cluster 1, with no significant difference found in stromal score (*P* = 0.1916; Figures 3A–D). In addition, TIMER algorithm indicated that the abundance of B cell (*P* = 0.0010), macrophage (*P* = 0.0036), dendritic cell (*P* < 0.0001) and T cell.CD4 (*P* = 0.0022) in cluster 2 was significantly higher than cluster 1, while the abundance of neutrophil was significantly higher in cluster 1 (*P* = 0.0004), and no statistical significance was detected with respect to T cell.CD8 (*P* = 0.6407; Figure 3E). Moreover, as illustrated in the heatmap (Figure 3F), immune landscape made by ssGSEA algorithm differed significantly between cluster 1 and cluster 2, with a relatively low immune status in cluster 1. Besides, statistical analysis demonstrated that except for eosinophils, mast cell, neutrophil and memory B cell, the other 24 types of cells were significantly higher in cluster 2 than those in cluster 1 (Figure 3G). These results demonstrated that the TIME and immune status of the two molecular subtypes differed significantly.

### DEG and Functional Analyses

**FIGURE 4:**
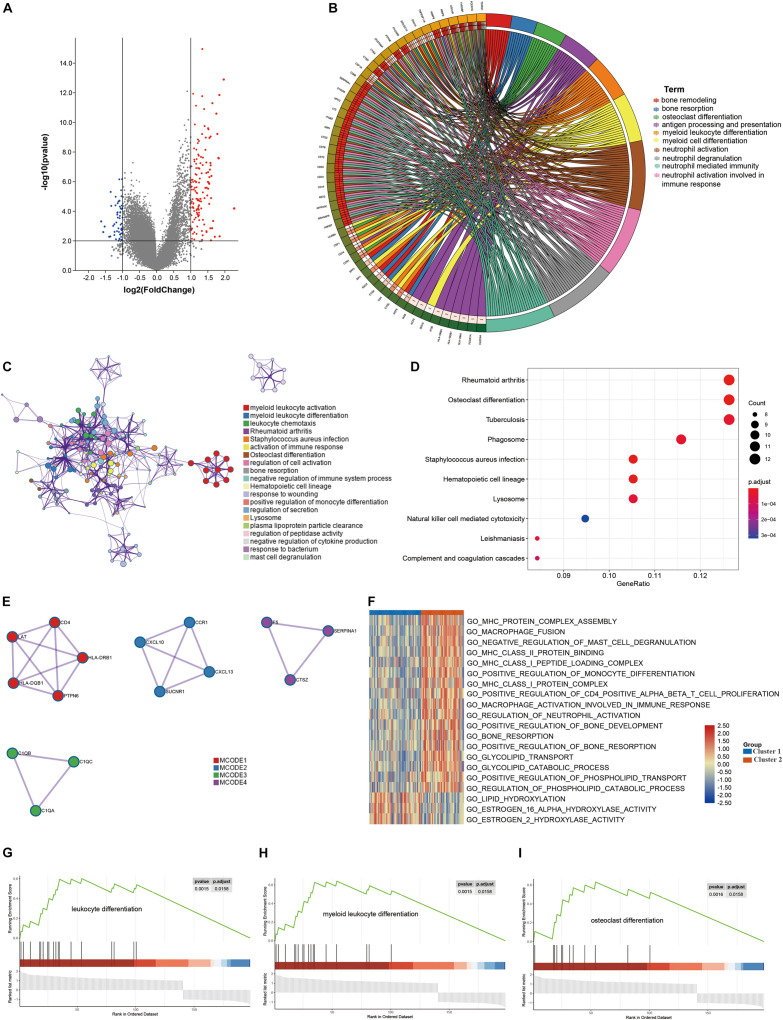
Differentially expressed genes (DEGs) analysis and functional analyses. **(A)** Volcano plot showing the DEGs between the two subgroups, **(B,C)** circle plot and network visualizing the biological processes enriched by gene ontology (GO) analysis, **(D)** bubble diagram showing the signaling pathways enriched by Kyoto Encyclopedia of Genes and Genomes (KEGG) analysis, **(E)** PPI analysis of DEGs, **(F)** heatmap illustrating the result of GSVA analysis, **(G–I)** GSEA plots visualizing the result of GSEA analysis.

DEGs between the two clusters were identified and functional analyses were performed to explore the underlying signaling mechanisms. A total of 198 DEGs were detected, of which 58 genes were downregulated and 140 genes were upregulated in cluster 2, as compared with cluster 1 (Figure 4A). GO enrichment analysis revealed that the DEGs were enriched in bone remodeling- and immune-related biological processes, including bone remodeling, bone resorption, antigen processing and presentation, immune cell differentiation and activation (Figures 4B,C). Meanwhile, some crucial molecular functions and cellular components were also enriched (Supplementary Figure 2). Similarly, KEGG enrichment analysis also identified some signaling pathways associated with bone remodeling and immune, including osteoclast differentiation, natural killer cell mediated cytotoxicity, and complement and coagulation cascade (Figure 4D). PPI analysis identified four sub- models, all of which were closely associated with tumor development and immunity, indicating that immune may be associated with the contribution of lipid metabolism to osteosarcoma (Figure 4E). To further explore the relationship between the enriched pathways and the prognosis of osteosarcoma patients, we performed GSVA and GSEA analyses to evaluate the relative expression difference of the pathways in the two clusters. GSVA analysis enriched a lot of differentially expressed pathways, which was visualized by the heatmap (Figure 4F). In comparison to cluster 2, the expression of pathways associated with immune, bone remodeling and lipid catabolic process were significantly lower in the cluster 1, whereas the expression of lipid hydroxylation associated pathways were significantly higher. Consistently, GSEA analysis revealed that leukocyte differentiation, myeloid leukocyte differentiation and osteoclast differentiation expressed lowly in cluster 1 (Figures 4G–I). All these results demonstrated that expression of LMRG was correlated with dysregulation of immune and bone remodeling, which may be involved in the poor prognosis of osteosarcoma patients.

### Development of Risk Model Based on LMRG in the Training Cohort

**FIGURE 5:**
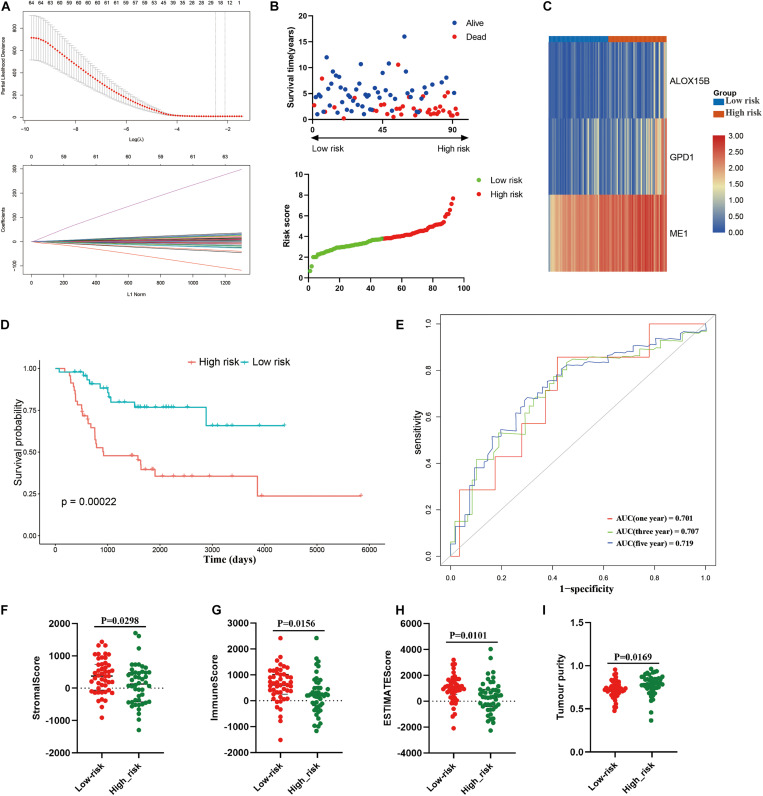
Construction of risk model in the training cohort. **(A)** LASSO analysis with minimal lambda, **(B)** distribution of survival status and risk score of osteosarcoma patients in the high and low risk groups, **(C)** heatmap illustrating the expression of the three candidate genes in the two groups, **(D)** survival curve of the osteosarcoma patients in the two groups, **(E)** time-dependent ROC curve of the risk model, **(F–I)** stomal score, immune score, ESTIMATE score and tumor purity calculated by ESTIMATE algorithm.

Then, a risk signature model was constructed to assess the prognostic prediction value of LMRGs in osteosarcoma. LASSO analysis was conducted to screen potential genes for establishing risk model, and 24 genes were filtered with optimal lambda value (Figure 5A). Based on the genes generated from LASSO analysis, multivariate Cox analysis identified three genes, *ME1*, *GPD1*, and *ALOX15B*, to construct the risk model. All of the three genes were risk genes with a hazard ratio of over 1, and Kaplan–Meier analysis demonstrated that all of these genes were independently prognostic marker of osteosarcoma patients (Supplementary Figure 3). The established risk model successfully classified the osteosarcoma patients into high risk and low risk groups (Figure 5B). As shown in Figure 5C, patients in the high risk group trended to expressed the three candidate genes higher than those in the low risk group. Patients in the low risk group had a better overall survival than those in the high risk group (Figure 5D). Regarding the model diagnosis of the risk model, ROC curve (Figure 5E) and residual plot (Supplementary Figure 4) showed acceptable assessment result. Time dependent ROC analysis indicated that the constructed risk model exhibited precise predictive capacity over a period of 5 years, and the area under curve (AUC) of the ROC curve for 1, 3, and 5 years was 0.701, 0.707, and 0.719, respectively (Figure 5E). Finally, ESTIMATE algorithm was performed to evaluate the TIME of the two groups, and the result revealed that compared with the high risk group, the stromal score (*P* = 0.0298), immune score (0.0156), ESTIMATE score (*P* = 0.0101) were significantly higher in the low risk group (Figures 5F–H), while the tumor purity was significantly lower (Figure 5I). These results suggested that the constructed risk model possessed potent potential for prognosis prediction of osteosarcoma patients, and it was significantly correlated with TIME in osteosarcoma.

### Independence of the Constructed Risk Model

**FIGURE 6:**
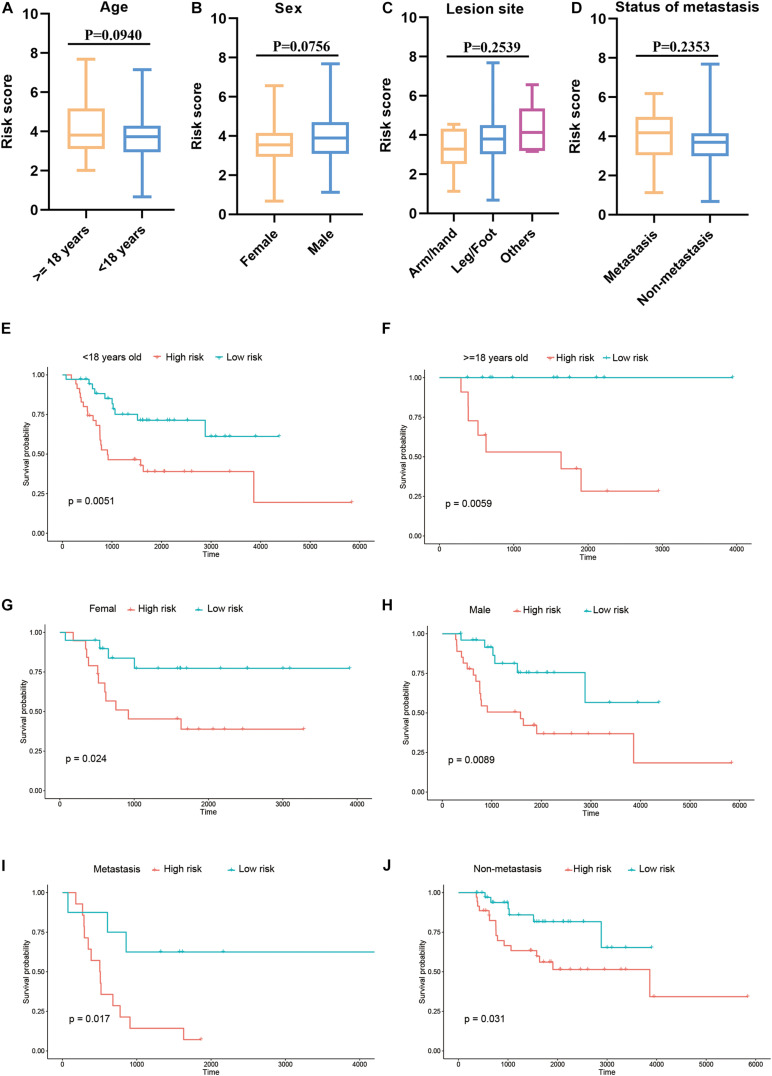
Association of risk score and clinical characteristics **(A–D)**. No significant difference was identified in patients with different age **(A)**, sex **(B)**, lesion site **(C)** and metastasis status **(D)**. Independence analysis of the risk model **(E–J)**. Survival curve of patients regrouped according to age **(E,F)**, sex **(G,H)**, and metastasis **(I,J)**.

Furthermore, we explored the association between the risk score and clinical features, and evaluated the independence of the constructed risk model via subgroup analysis and regression analyses. No significant difference was detected between patients with different age (Figure 6A), sex (Figure 6B), lesion site (Figure 6C) and metastasis status (Figure 6D) regarding risk score, indicating that there was no association between risk score and clinical characteristics (Figures 6A–D). Besides, when the patients were regrouped according to age (Figure 6E), sex (Figure 6F), and metastasis status (Figure 6G), the risk model still exhibited potent predictive performance and those patients with lower risk score enjoyed better prognosis. Moreover, univariate/multivariate Cox regression analyses revealed that the constructed risk model was independent predictive marker of the prognosis of osteosarcoma patients (Tables 2, 3). These results demonstrated that the constructed risk model had excellent independence in predicting the prognosis in osteosarcoma.

**TABLE 2:** Univariate analysis of risk score and characteristics in training cohort.

Variates	Coefficient	HR	HR 95%CI (lower)	HR 95%CI (upper)	*p*-value
Risk score	0.52465566	1.68987686	1.309302499	2.1810726	5.58E−05
Sex	−0.0203184	0.97988661	0.510247494	1.8817883	0.951337
Age	−0.0233226	0.97694732	0.914836448	1.0432751	0.486496
Metastasis	1.33251557	3.79056685	1.978018385	7.2640361	5.94E−05
lesion site	1.36256868	3.90621424	1.225603012	12.449798	0.021226

*HR, hazard ratio; CI, confidence interval.*

**TABLE 3:** Multivariate analysis of risk score and characteristics in training cohort.

Variates	Coefficient	HR	HR 95%CI (lower)	HR 95%CI (upper)	*p*-value
Risk score	0.554978	1.741903	1.324072765	2.29158578	7.31E−05
Sex	−0.16642	0.846691	0.426467921	1.680985208	0.63435
Age	−0.01876	0.981413	0.910831832	1.057463256	0.622222
Metastasis	1.4284	4.17202	2.090665046	8.325459388	5.08E−05
Lesion site	0.877566	2.405038	0.902139129	6.411659127	0.079412

### Risk Model Was Correlated With TIME and Prognosis in Osteosarcoma in the Verification Cohort

**FIGURE 7:**
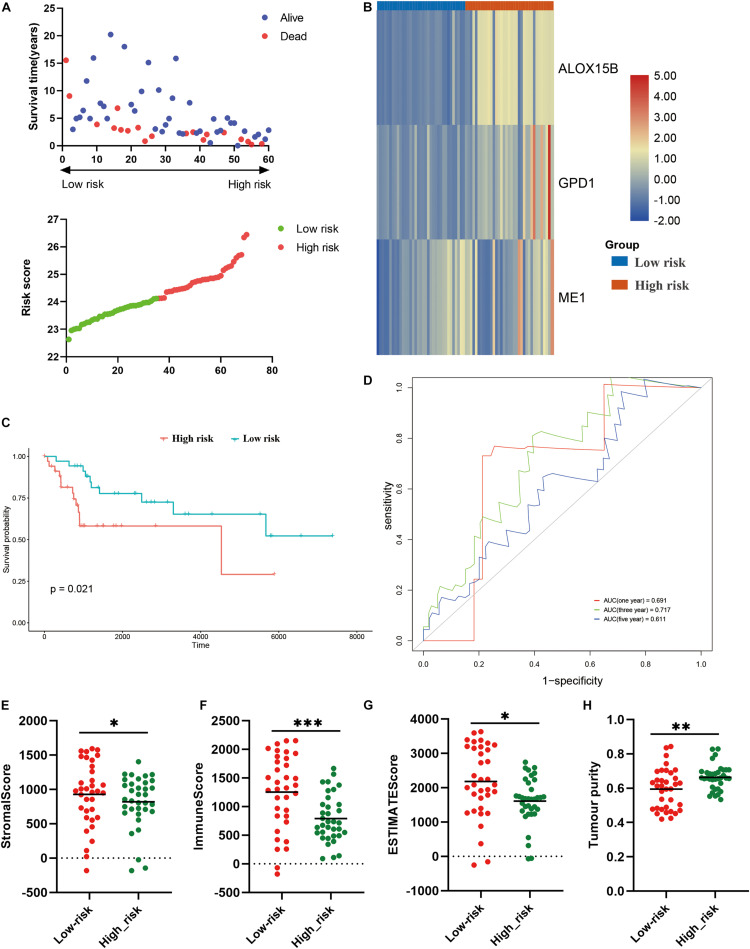
Validation of the constructed risk model in the verification cohort. **(A)** distribution of survival status and risk score, **(B)** heatmap illustrating the expression of three candidate genes in the verification cohort, **(C)** survival curve of the patients in the high and low risk groups in the verification cohort, **(D)** ROC curve of the risk model in the verification cohort, **(E–H)** stromal score, immune score, ESTIMATE score and tumor purity calculated by ESTIMATE algorithm. *p < 0.05; **p < 0.01; ***p < 0.001.

Thereafter, we further validate the established prognostic risk score model in the verification cohort. According to above-mentioned formula, the osteosarcoma patients in the verification cohort were stratified into high risk or low risk groups (Figure 7A). The expression of the three candidate genes were shown via the heatmap (Figure 7B). Survival analysis revealed that patients in the high risk group had poorer prognosis (*P* = 0.021; Figure 7C). ROC analysis indicated that the risk model exhibited the best prediction accuracy in predicting 3-year survival (Figure 7D). We also explore the association between the risk model and TIME. Same to the training cohort, in comparison to the high risk group, the stromal score (*P* < 0.05), immune score (*P* < 0.001), ESTIMATE score (*P* < 0.05) were significantly higher in the low risk group (Figures 7E–G), while the tumor purity was significantly lower (*P* < 0.01; Figure 7H). These results demonstrated that the established risk model was correlated with TIME and prognosis in osteosarcoma in the verification cohort.

### Construction and Calibration of an Integrated Monogram

**FIGURE 8:**
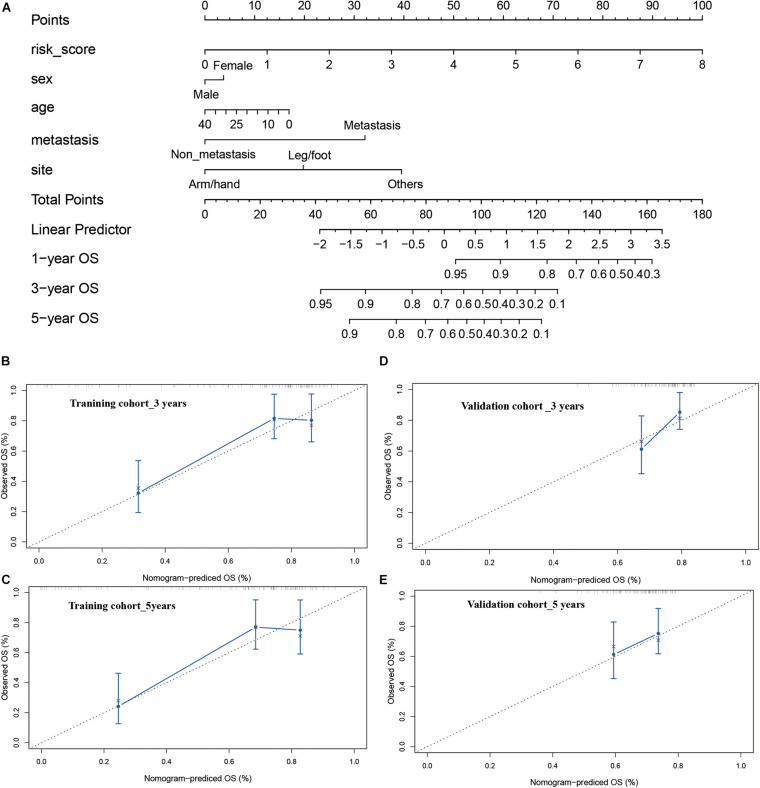
Construction and calibration of nomogram. **(A)** nomogram integrating risk score and clinical features, **(B,C)** calibration of the nomogram at 3 and 5 years in the training cohort, **(D,E)** calibration of the nomogram at 3 and 5 years in the verification cohort.

Finally, a nomogram integrating the risk model and clinical features were constructed to predict the prognosis of the osteosarcoma patients more precisely. The constructed nomogram was shown in Figure 8A, risk score and pathological characteristics were endowed a specific score basing on their contribution on the prognosis in osteosarcoma. Then we validated the nomogram in the training and verification cohort. In terms of the model diagnosis of the nomogram, C-index, the calibration curve (Figures 8B–D) and decision curve analysis (Supplementary Figure 5) suggested the acceptable accuracy. The C-index for the nomogram in the training cohort reached 0.7520 (95%CI: 0.7078–0.7962). The observed overall survival matched well with the actual survival at 3 and 5 years in the training cohort (Figures 8B,C), and similar result was also observed in the verification cohort (Figures 8D,E). These results demonstrated that the integrated nomogram could predict the prognosis of osteosarcoma patients accurately.

All of these findings revealed that lipid metabolism dysregulation may lead to disorder of TIME and bone remodeling, resulting in poor prognosis. The constructed risk model based on LMRGs could predict the prognosis of osteosarcoma patients reliably and accurately.

## Discussion

Osteosarcoma was the most common primary malignant tumor of bone in children and adolescent (Niu et al., 2020). Despite of the booming development of multiple treatment strategy, the 5-year survival of osteosarcoma patients has remained stagnant over the past 35 years, and it is urgent to develop effective risk stratification approach and individualized targeting treatment strategy (Kansara et al., 2014; Negri et al., 2019). In the present study, we identified two molecular subtypes, which exhibited significantly different lipid metabolism landscapes. Immune analyses indicated that patients with poor prognosis were in relatively low immune status, and possessed lower immune score and ESTIMATE score whereas higher tumor purity, as compared with patients with better prognosis. Further functional analyses revealed that upregulation of lipid hydroxylation was implicated with poor immunity and bone remodeling. Moreover, we established a prognostic risk model based on LMRGs, which predicted the prognosis of osteosarcoma patients precisely. Our results may facilitate the development of targeting therapy for osteosarcoma and help the clinicians to make more rational treatment decisions.

Consensus clustering was a reliable approach to classified samples into different subgroups based on the gene expression matrix. According to the LMRGs expression matrix of osteosarcoma patients, firstly, we identified two molecular subgroups via consensus clustering, which also had significantly different overall survival. Then immune and function analyses were performed to explore the role of lipid metabolism in osteosarcoma successively.

As mentioned previously, TIME plays a crucial role in the prognosis of patients, since tumor progression is associated with the modification of the surrounding stroma, with immune cells being the pivotal components of tumor stroma (Hinshaw and Shevde, 2019). In addition, aberrant metabolism status of tumor cells would result in metabolic variation of TIME. ESTIMATE algorithm was an innovative method to infer the tumor purity, as well as the fraction of immune and stromal cells in tumor according to gene expression value (Yoshihara et al., 2013). Immune scores generated from ESTIMATE algorithm displayed immune components in tumor samples quantitatively, reflecting the TIME. Tumor purity was defined as the proportion of malignant cells in tumor tissue, which was closely correlated with prognosis (Yoshihara et al., 2013; Aran et al., 2015; Li et al., 2017). Previously, Hong et al. (2020) and Zhang et al. (2020) have demonstrated that high immune score and low tumor purity were linked with poor prognosis in osteosarcoma. Therefore, we applied ESTIMATE to determinate the TIME of the two subgroups. Our result indicated that these patients with better prognosis had higher immune score and lower purity, which was consistent with previous reports. Furthermore, we applied another two methods, TIMER and ssGSEA, to assess the immune status of the two molecular subgroups. TIMER was a web tool which facilitated the quantification of six tumor-infiltrating immune subsets (Li et al., 2017). TIMER analysis revealed that the abundance of five out of six immune cells was significantly lower in the cluster 1, which was in concert with the result of ESTIMATE and indicated that the immune landscape was downregulated in cluster 1. The ssGSEA analysis outlined the abundance of 29 immune-related cells, and the result suggested that patients in cluster 1 were in relatively low immune status, further confirming the result of ESTIMATE and TIMER. Taken together, we could assume that low immune score and immune status were implicated with unfavorable prognosis reasonably.

Next, functional analyses between the two subgroups were conducted to explore the underlying biological mechanisms. Based on the identified DEGs, GO analysis, KEGG analysis and PPI analysis synergistically suggested that dysregulation of immunity and bone remodeling may mediate the role of lipid metabolism on the tumorigenesis and progression of osteosarcoma. However, the detailed relationship between lipid metabolism and aberrant immunity and bone remodeling remained unclear. Therefore, we performed GSVA and GSEA analyses to further elucidate the underlying mechanisms. Through GSVA, the activity of signaling pathways in each sample was calculated according to the gene expression level, and the variation over different groups could be estimated (Hänzelmann et al., 2013). The GSVA heatmap result revealed that the activity of lipid catabolism, bone remodeling, immune system development and activation was impeded in cluster 1, whereas lipid hydroxylation was enhanced. GSEA analysis was a canonical method for integrating gene expression information, through which the expression tendency of gene sets in different groups were clarified directly (Subramanian et al., 2005). In this study, GSEA result revealed relatively low expression of immune cells differentiation and osteoclast differentiation in cluster 1. These results indicated that downregulated lipid catabolism and upregulated lipid hydroxylation were implicated with low immune status and poor bone remodeling.

Synthesizing above findings, we could deduce reasonably that dysregulation of lipid metabolism, including hydroxylation upregulation and catabolism downregulation, resulted in the impairment of TIME and bone remodeling, thereby leading to the poor prognosis in osteosarcoma. As mentioned above, lipid metabolism reprogramming was recognized as new hallmark of tumor malignancy. Over the past years, lipid metabolic abnormalities of tumor have gained increasing attention (Maan et al., 2018). Targeting aberrant pathways of lipid metabolism is a promising strategy for antitumor therapy. For example, anti-tumor drugs based on the hydroxylated lipid has been widely used for tumor treatment clinically (Király et al., 2013). In this study, upregulated lipid hydroxylation was found to be associated with poor survival. Among the multiple modification of lipids, hydroxylation was a specific approach during which oxygens were added onto the lipids in the manner of hydroxyl through radical oxygen species (ROS) or non-radical oxidants (Spickett, 2020). As one of the activated radicals of biological systems, hydroxyl radical was prone to induced lipid peroxidation (Spickett, 2020). Because lipids were critical components of multiple membranes and distributed widely (Cheng et al., 2018), lipid peroxidation usually occurred when polyunsaturated fatty acids (PUFAs) were attacked by ROS, which would impair the structure and/or function of membranes subsequently (Yoshida et al., 2013). Lipid peroxidation resulted in the formation of aldehydes with high reactivity (Erejuwa et al., 2013), which further attack the components of cellular membrane, such as lipids and proteins (Höhn and Grune, 2013). Meanwhile, some other products of lipid peroxidation, including malondialdehyde, 4-hydroxynonenal and acrolein, also bound to the amino acid residues of protein covalently via Michael addition, damaging the structure and function of the residues (Fukuda et al., 2009). All of these could explain the contribution of upregulated hydroxylation to the poor prognosis of cancer patients in a certain extent. On the other hand, catabolism downregulation reduced the consumption of lipids, and as a result, accumulative lipids were stored in tumor cells and surrounding cells. The influence of accumulated lipid due to lipid metabolism abnormalities on the tumor-microenvironment dendritic cells may also account for the poor prognosis partly. Because previous studies reported that lipid-laden dendritic cells were unable to present tumor-associated antigens (Herber et al., 2010). And abnormal lipid accumulation inhibited the capacity of dendritic cells to facilitate anti-tumor T cells (Cubillos-Ruiz et al., 2015). This also expounded why the downregulated lipid metabolism led to lower immune score and immune status. Besides, the accumulative lipid may result in poor prognosis via facilitating metastasis which was a critical factor for tumor progression, since the metastatic potential of tumor cells was positively correlated with intracellular lipid storage (Maan et al., 2018).

To further validate the effect of lipid metabolism disorder on the TIME in osteosarcoma and explore the prognostic value of LMRGs in osteosarcoma patients, we constructed a prognostic risk model based on LMRGs and verified it in the validation cohort. The three genes used for establishing risk model in this study have been demonstrated to be closely associated with development and progression of tumors. *ME1* encodes malic enzyme 1 (ME1), which catalyzes the transformation of malate to pyruvate and promotes the formation of NADPH concomitantly. Prior studies reported that ME1 mediated the lipid metabolism through participating in lipid biosynthesis basically (Simmen et al., 2020). Numerous researches have provided the evidence of the pro-oncogenic role of ME1 in multiple tumors (Simmen et al., 2020). *ALOX15B* (Arachidonate 15-Lipoxygenase Type B) has been demonstrated as risk genes in colorectal cancer and lung squamous cell carcinoma (Yuan et al., 2020; Kim et al., 2021). *GPD1* encodes glycerol-3-phosphate dehydrogenase 1 (GPD1), which played a pivotal role in lipid metabolism through catalyzing the transformation of NADH and dihydroxyacetone phosphate to NAD+ and glycerol-3-phosphate reversibly (Yoneten et al., 2019). Studies suggested that *GPD1* was closely related to prognosis of gastrointestinal cancer, breast cancer, and glioblastoma (Xie et al., 2020; Xia et al., 2021). Survival analysis revealed that no matter in the training cohort or the verification cohort, the established risk model exhibited potent predictive performance for the survival of osteosarcoma patients. And significantly lower stromal score, immune score and higher tumor purity were accompanied with poor survival. Moreover, independence analysis and subgroup analysis suggested that the LMRGs-based risk model could predict the prognosis independently in osteosarcoma, regardless of their age, sex and metastasis condition. Finally, a nomogram integrating the risk score and clinical features was also established and calibrated, and it showed considerable property for predicting the survival. All these results confirmed the prognostic prediction role of LMRGs in osteosarcoma and correlation between aberrant lipid metabolism and TMIE disorder.

Over the recent decades, radiotherapy and chemotherapy for tumors have grew boomingly. However, the 5-year survival of osteosarcoma remained unsatisfactory (Ritter and Bielack, 2010; Kansara et al., 2014; Heng et al., 2020). It is imperative to develop effective methods to classify the patients according their risk score, and conduct reasonable individualized and targeted therapy. Bioinformatic analysis based on sequencing RNA data was a feasible approach for risk stratification and targeted-gene identification. Although researchers have constructed risk model based on tumor microenvironment, immune cell infiltrating and energy metabolism (Chen Y. et al., 2020; Wen et al., 2020; Zhang et al., 2020; Zhu et al., 2020) in osteosarcoma, our study exhibited unique merits compared with previous studies. Firstly, our work focused on the lipid metabolism of osteosarcoma patients, and identified two molecular subgroups with significantly different prognosis and immune status via consensus clustering. Secondly, we explored the biological mechanisms according to the result of clustering and elucidated the underlying mechanism partly. Thirdly, we clarified the influence of lipid metabolism on TIME and prognosis. Last but not least, this work integrated two GEO datasets as a verification cohort, which containing much more samples than previous studies. Our work would provide excellent theoretical instruction for further studies of osteosarcoma. Additionally, result of this study could promote the development of targeted therapy for osteosarcoma and help the clinicians make treatment strategy more rationally.

In general, in this study, we identified two molecular subtypes, cluster 1 and 2. In cluster 1, patients with poor prognosis showed TIME disorder including low immune score and high tumor purity, and the dysregulation of lipid catabolism was implicated with low immune status and aberrant bone remodeling. Risk model based on LMRGs could predicted the prognosis in osteosarcoma precisely, meanwhile, those patients with unfavorable survival in the high risk showed low immune score and high tumor purity. These results indicated that lipid metabolism landscape was correlated with TIME, and deserved considerable attention in determining treatment strategy for osteosarcoma patients, which was a potential target for individualized treatment.

There were some drawbacks in our study which should be notified when generalizing the conclusion. Firstly, due to the absence of information about the progression of the osteosarcoma patients, such as tumor stages, we could not demonstrate the role of LMRG in the development of osteosarcoma. Secondly, our results generated from bioinformatics analysis, which was not further validated by experiments. Thirdly, the data used in this study was downloaded from open database instead of our cohort. The nature of low evidence level of retrospective research still remained, and more prospective studies are needed to be conducted to further confirm the prognostic value of LMRGs in osteosarcoma.

## Conclusion

In conclusion, in the present study, two molecular subtypes were identified based on LMRGs in osteosarcoma via consensus clustering. Immune analysis and functional analyses revealed that dysregulation of lipid metabolism would impede the immune system and bone remodeling, thereby resulting in poor prognosis. Our work could shed a novel light on the development of new targeting drugs, provide theoretical support for individualized therapy, and facilitate the risk stratification of osteosarcoma patients.

## Data Availability Statement

The datasets presented in this study can be found in online repositories. The names of the repository/repositories and accession number(s) can be found in the article/Supplementary Material.

## Author Contributions

YH and PL conceived the original ideas of this manuscript and reviewed the finished manuscript and executed supervision throughout the process. HQ executed the data collection and data analysis. HQ and TL prepared the manuscript, tables, and figures. All authors have read and approved the manuscript.

## Conflict of Interest

The authors declare that the research was conducted in the absence of any commercial or financial relationships that could be construed as a potential conflict of interest. The reviewer JZ declared a shared affiliation with all authors to the handling editor at the time of review.

## Footnotes

1 https://ocg.cancer.gov/programs/target

2 https://www.ncbi.nlm.nih.gov/geo/

3 https://cistrome.shinyapps.io/timer/

4 https://www.gseamsigdb.org/gsea/msigdb/index.jsp

5 https://metascape.org
