# Supplementary material for: Expression of Lipid-Metabolism Genes Is Correlated With Immune Microenvironment and Predicts Prognosis in Osteosarcoma
Source: Front Cell Dev Biol. 2021 Apr 16;9:673827. doi: 10.3389/fcell.2021.673827 (PMC8085431; doi:10.3389/fcell.2021.673827)
Supplement: Supplementary file 1 [file Table_1.DOCX]

**Supplementary table 1**. Significant genes generated in Univariate Cox regression analysis

| Genes | Coefficient | HR | HR 95%CI (lower) | HR 95%CI (upper) | p-Value |
| --- | --- | --- | --- | --- | --- |
| MED27 | -0.90012 | 0.406521 | 0.261371 | 0.632279 | 6.49E-05 |
| ASAH2 | 0.863137 | 2.370585 | 1.450048 | 3.875507 | 0.000578 |
| PIP5K1C | -0.83998 | 0.431719 | 0.259513 | 0.718198 | 0.001218 |
| PLA2G4F | 0.809567 | 2.246936 | 1.375031 | 3.671715 | 0.001234 |
| MED18 | -0.70946 | 0.491911 | 0.317866 | 0.761254 | 0.001451 |
| CRAT | -0.65879 | 0.517479 | 0.342436 | 0.781997 | 0.001764 |
| HSD11B2 | 0.474947 | 1.607929 | 1.176484 | 2.197595 | 0.002886 |
| GM2A | -0.69596 | 0.498598 | 0.314409 | 0.790687 | 0.003094 |
| AMACR | -0.66165 | 0.515997 | 0.332525 | 0.800701 | 0.003163 |
| ALOX15B | 0.904224 | 2.470013 | 1.337533 | 4.561357 | 0.003862 |
| GLB1 | -0.64691 | 0.52366 | 0.337203 | 0.813219 | 0.003969 |
| PPARG | -0.42245 | 0.655439 | 0.487647 | 0.880966 | 0.005111 |
| SCD5 | -0.53443 | 0.586005 | 0.401572 | 0.855145 | 0.00558 |
| SAR1B | -0.73632 | 0.478874 | 0.281947 | 0.813344 | 0.006442 |
| GDE1 | -0.61094 | 0.542839 | 0.347042 | 0.849103 | 0.007437 |
| VAC14 | -0.64592 | 0.524181 | 0.326019 | 0.842789 | 0.007679 |
| PLIN3 | -0.58857 | 0.555122 | 0.357845 | 0.861157 | 0.008609 |
| TNFRSF21 | -0.38123 | 0.683021 | 0.512502 | 0.910274 | 0.009282 |
| GLIPR1 | -0.45967 | 0.631492 | 0.446632 | 0.892865 | 0.009289 |
| CYP2C8 | 0.635376 | 1.887733 | 1.168112 | 3.050678 | 0.009474 |
| ACP6 | -0.44456 | 0.641103 | 0.457407 | 0.898573 | 0.009857 |
| PITPNM3 | 0.509083 | 1.663765 | 1.12788 | 2.454261 | 0.010267 |
| PLD3 | -0.48614 | 0.614997 | 0.422667 | 0.894844 | 0.011066 |
| NEU1 | -0.55323 | 0.575089 | 0.374579 | 0.882931 | 0.011433 |
| CYP27A1 | -0.37902 | 0.68453 | 0.510276 | 0.918292 | 0.01145 |
| BDH2 | -0.62117 | 0.537316 | 0.331656 | 0.870506 | 0.011626 |
| OXCT2 | 0.464244 | 1.590811 | 1.102086 | 2.296263 | 0.013174 |
| PI4K2A | -0.69132 | 0.500916 | 0.289497 | 0.866732 | 0.013465 |
| CYP2R1 | -0.65555 | 0.519158 | 0.308212 | 0.874479 | 0.013735 |
| MAPKAPK2 | -0.67596 | 0.50867 | 0.294242 | 0.879362 | 0.015509 |
| GPX1 | -0.37988 | 0.68394 | 0.502186 | 0.931475 | 0.015937 |
| ABHD5 | -0.59387 | 0.552186 | 0.34058 | 0.895263 | 0.016009 |
| CSNK2B | -0.55582 | 0.573599 | 0.363498 | 0.90514 | 0.016931 |
| CPTP | -0.59307 | 0.552631 | 0.337899 | 0.903823 | 0.018135 |
| PRKACB | -0.48259 | 0.617182 | 0.412932 | 0.922461 | 0.018593 |
| ACOT1 | -0.54724 | 0.578544 | 0.366015 | 0.914478 | 0.019145 |
| ACER2 | 0.74222 | 2.100594 | 1.126675 | 3.916385 | 0.019532 |
| FAAH | 0.360825 | 1.434513 | 1.059397 | 1.94245 | 0.019646 |
| CBR1 | -0.47717 | 0.620538 | 0.414947 | 0.927992 | 0.020129 |
| PTPMT1 | -0.57923 | 0.560328 | 0.343725 | 0.913425 | 0.020171 |
| PIK3R2 | -0.47868 | 0.619598 | 0.413259 | 0.928963 | 0.020527 |
| GPD1 | 0.34157 | 1.407154 | 1.051802 | 1.882564 | 0.021445 |
| PLA2G12A | -0.46638 | 0.627267 | 0.421302 | 0.933924 | 0.021642 |
| HMGCL | -0.61539 | 0.540429 | 0.317077 | 0.921112 | 0.023697 |
| DGKZ | -0.50581 | 0.603015 | 0.388734 | 0.935414 | 0.023945 |
| ALOX5AP | -0.30078 | 0.740239 | 0.568454 | 0.963937 | 0.025576 |
| SLC27A5 | 0.41788 | 1.518739 | 1.047916 | 2.2011 | 0.027302 |
| MED21 | -0.4988 | 0.607262 | 0.389759 | 0.94614 | 0.027477 |
| ARNTL | -0.57538 | 0.56249 | 0.335474 | 0.943128 | 0.029108 |
| FDPS | 0.551492 | 1.735841 | 1.056007 | 2.853338 | 0.02964 |
| ACADVL | 0.514052 | 1.672052 | 1.050774 | 2.660665 | 0.030088 |
| CYP2C19 | 1.530613 | 4.621006 | 1.132362 | 18.85767 | 0.032908 |
| ME1 | 0.337435 | 1.401349 | 1.024828 | 1.916201 | 0.034551 |
| TNFAIP8 | -0.49486 | 0.609653 | 0.384385 | 0.96694 | 0.035481 |
| PIKFYVE | 0.455612 | 1.577139 | 1.029543 | 2.41599 | 0.036281 |
| HSD3B7 | -0.3081 | 0.734842 | 0.550336 | 0.981205 | 0.036745 |
| HTD2 | -0.47162 | 0.62399 | 0.398935 | 0.976008 | 0.038794 |
| SLC25A1 | -0.40716 | 0.665538 | 0.451649 | 0.980718 | 0.039552 |
| ACOT9 | -0.52638 | 0.59074 | 0.357419 | 0.97637 | 0.040049 |
| KPNB1 | -0.4097 | 0.663852 | 0.448232 | 0.983195 | 0.0409 |
| PLD2 | 0.406014 | 1.500824 | 1.014051 | 2.221263 | 0.042385 |
| PLA2G6 | 0.349889 | 1.418911 | 1.01201 | 1.989414 | 0.042438 |
| PTGR2 | 0.49949 | 1.647881 | 1.017154 | 2.669716 | 0.042452 |
| LRP2 | 0.432351 | 1.540876 | 1.013363 | 2.34299 | 0.043171 |
| ALAS1 | -0.4637 | 0.628951 | 0.400478 | 0.987767 | 0.044072 |
| TM7SF2 | 0.400984 | 1.493293 | 1.010695 | 2.206327 | 0.044075 |
| CD36 | 0.230676 | 1.259451 | 1.005671 | 1.577274 | 0.044514 |
| CYP11B2 | 4.400422 | 81.48528 | 1.113694 | 5962.007 | 0.044523 |
| UBE2I | -0.56637 | 0.567582 | 0.326283 | 0.987333 | 0.044953 |
| AGPAT3 | -0.40722 | 0.665498 | 0.445888 | 0.993272 | 0.046261 |
| PRKD2 | -0.56953 | 0.565789 | 0.322163 | 0.99365 | 0.047464 |
| OSBPL9 | 0.525638 | 1.691537 | 1.00527 | 2.846297 | 0.04773 |
| PIK3CG | -0.64159 | 0.526457 | 0.278132 | 0.996493 | 0.048752 |
| INPP4A | 0.391671 | 1.47945 | 1.000811 | 2.187 | 0.049527 |

HR, hazard ratio; CI, confidence interval.


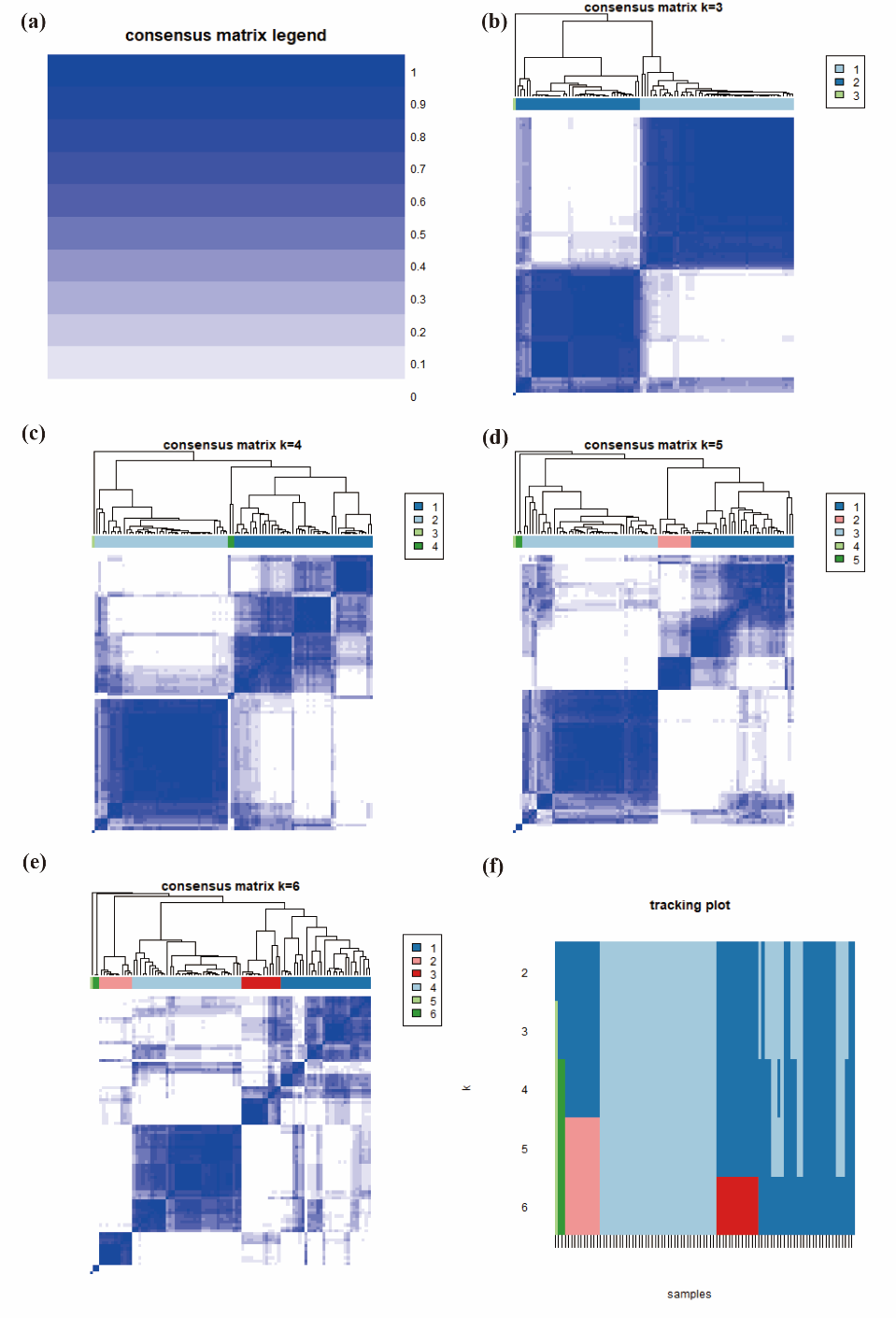
Supplemental figure 1. Consensus clustering of osteosarcoma patients.


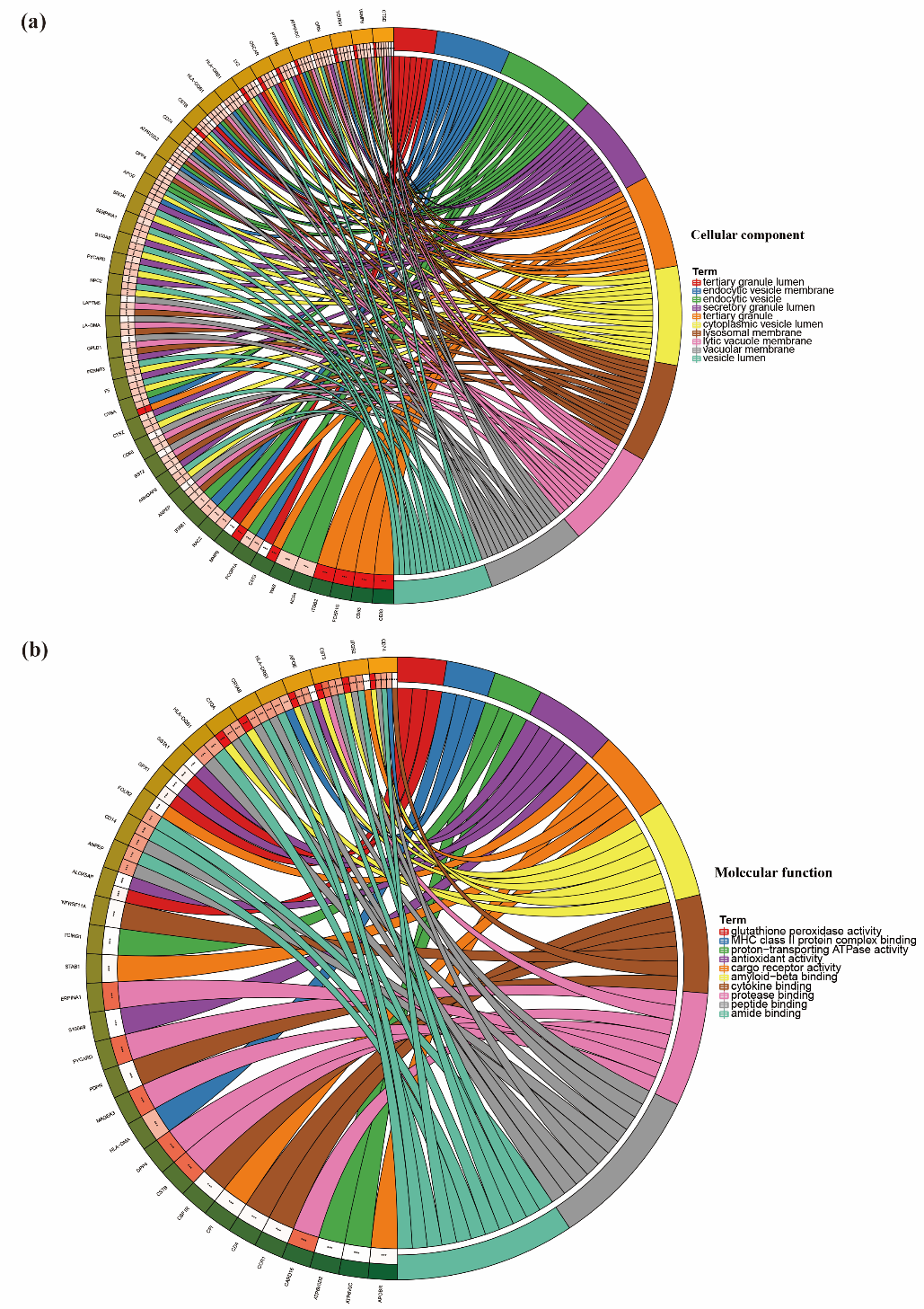


Supplemental figure 2. Gene ontology analysis of the DEGs between the two clusters. (a) cellular components enrichment analysis of the differentially expressed genes, (b) molecular function enrichment analysis of the differentially expressed genes.


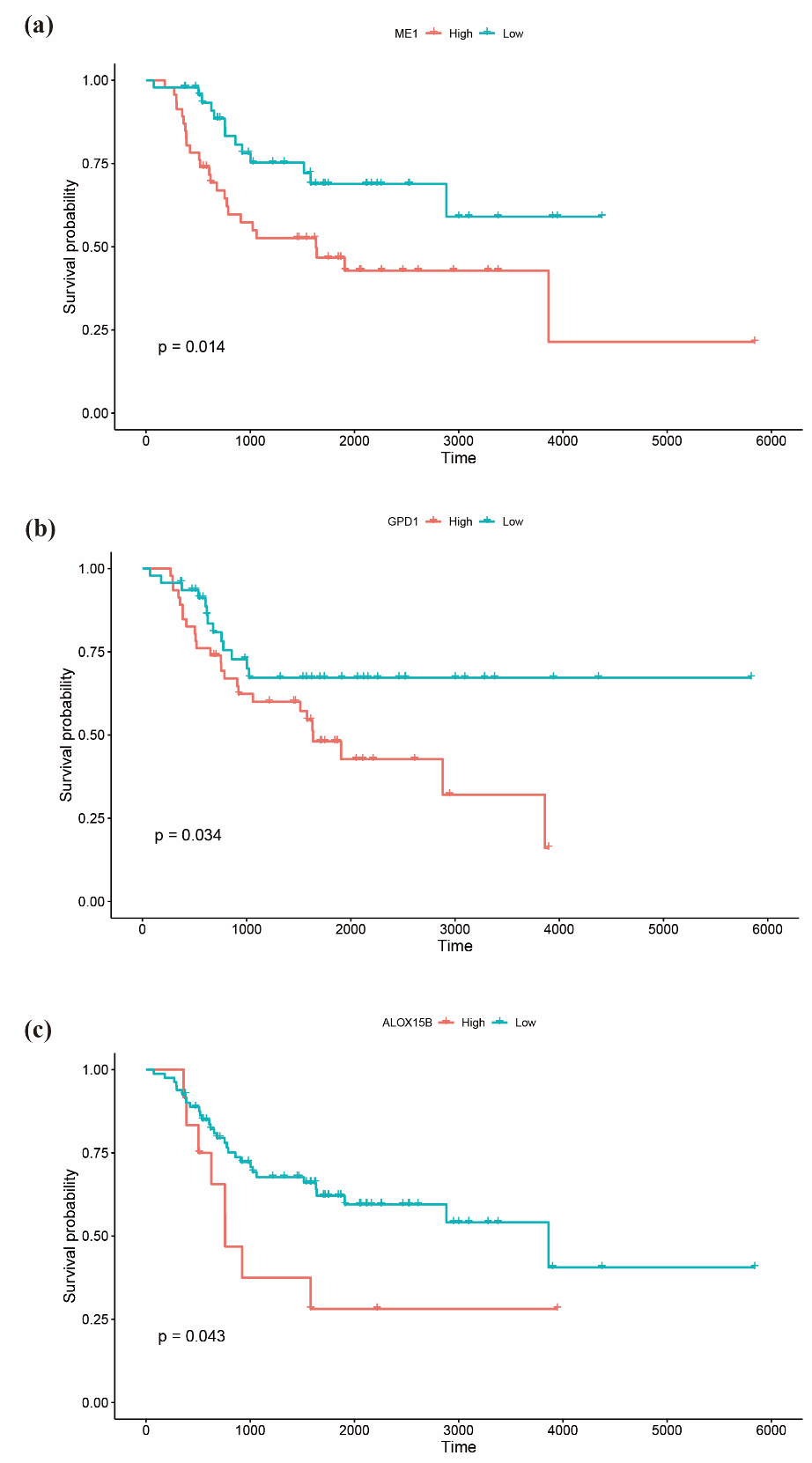


Supplemental figure 3. Survival analysis of the three candidate genes. All of ME1, GPD1 and ALOX15B were independently prognostic gene in osteosarcoma.


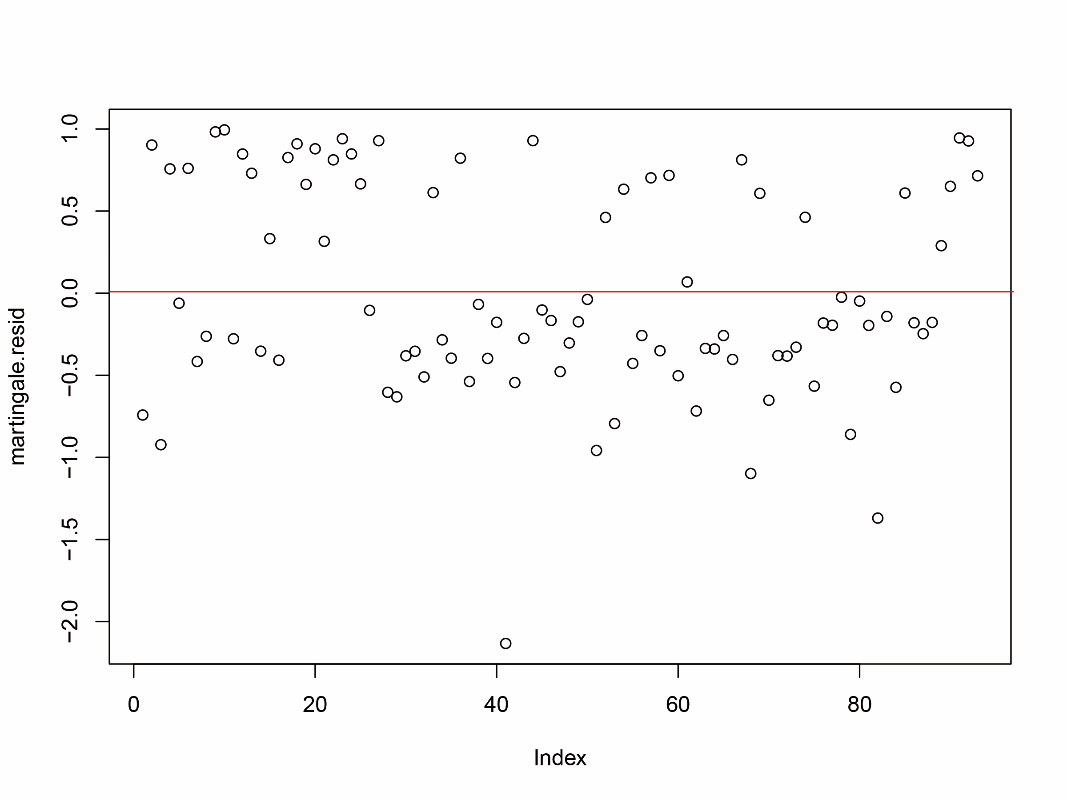
Supplemental figure 4. Residual plot of the constructed LMRGs-based risk model. Martingale residuals plot demonstrating that the patients distributed around zero randomly.


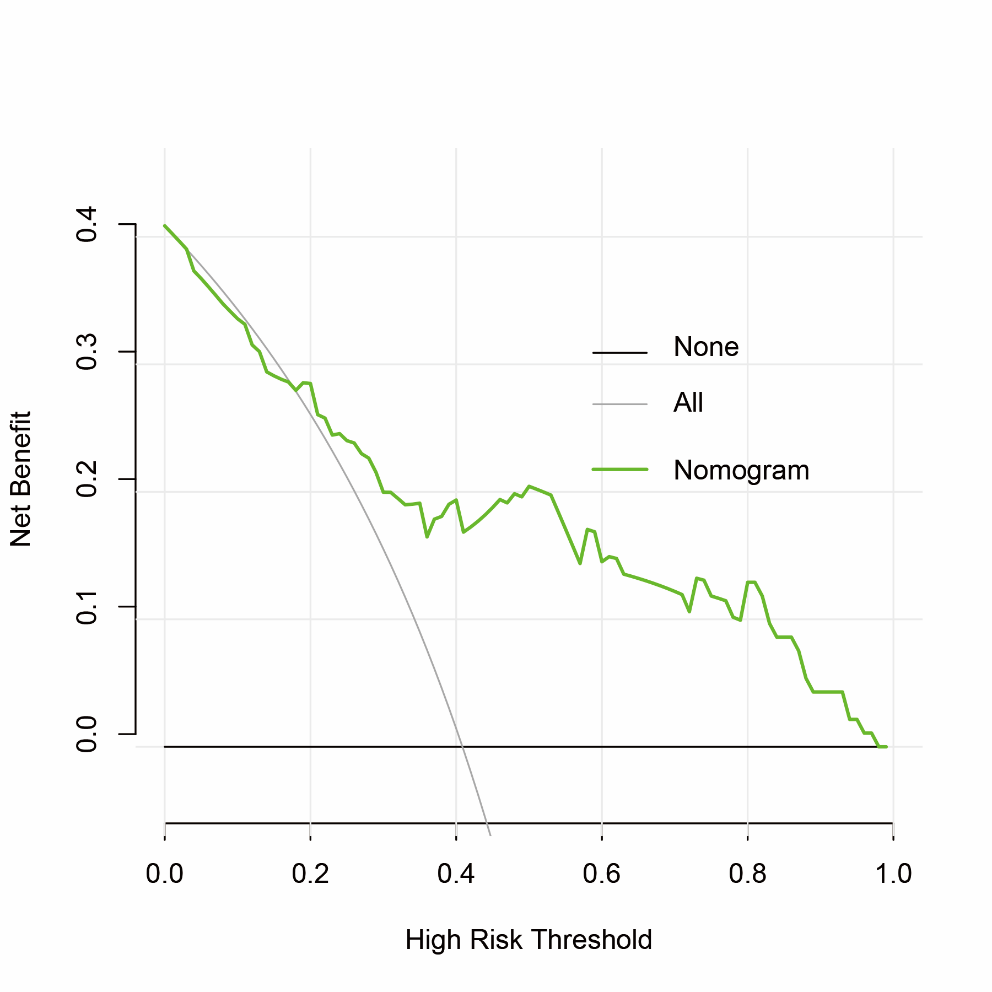
Supplemental figure 5. Decision curve of the nomogram demonstrating net benefit between the threshold probabilities of ∼20 and ∼95% for the nomogram predicting prognosis.
